# Macrophages play a key role in tissue repair and regeneration

**DOI:** 10.7717/peerj.14053

**Published:** 2022-09-29

**Authors:** Yajie Yu, Zhongyu Yue, Mengli Xu, Meiling Zhang, Xue Shen, Zihan Ma, Juan Li, Xin Xie

**Affiliations:** College of Life Science, Northwest University, Xi’an, Shaanxi, China

**Keywords:** Macrophage, Tissue repair, Regeneration, Cytokine, Polarization

## Abstract

Tissue regeneration after body injury has always been a complex problem to resolve for mammals. In adult mammals, the repair process after tissue injury is often accompanied by continuous and extensive fibrosis, which leads to scars. This process has been shown to severely hinder regeneration. Macrophages, as widely distributed innate immune cells, not only play an important role in various pathological processes, but also participate in the repair process before tissue regeneration and coordinate the regeneration process after repair. This review will discuss the various forms and indispensability of macrophages involved in repair and regeneration, and how macrophages play a role in the repair and regeneration of different tissues.

## Introduction

Tissue repair and regeneration are prerequisites for every living organism. The regenerative capacity of most vertebrates is limited if the complex tissue suffers severe physical damage (*e.g.*, surgical excision, stabbing, cryogenic injury, *etc.*), as they are not completely self-healing. Past studies have found that mammals such as mice possess powerful regenerative abilities during their neonatal stage. When cartilage, skin or nerve damage occur during the fetal stage, mammels rely on a unique and complex molecular mechanism to achieve complete tissue regeneration (Kishi et al., 2006; Yannas, Kwan & Longaker, 2007; Ribitsch et al., 2021a; Ribitsch et al., 2021b), but this amazing regenerative potential also disappears in adulthood (Sarig & Tzahor, 2017; Seifert & Muneoka, 2018; Yannas & Tzeranis, 2021). Research has now focused on understanding the necessary procedures for this hidden regenerative potential.

When the organism inevitably suffers from injuries or infection, the immune system responds immediately and targets the damaged surface to trigger an inflammatory response and simultaneously eliminate pathogens or microorganisms accompanying tissue damage (Zhang et al., 2010). Inflammation is the first reaction before tissue repair. Before inflammation occurs, it needs to recruit, proliferate, and activate various bone marrow hematopoietic cells or non-bone marrow hematopoietic cells, including neutrophils, macrophages, natural killer (NK) cells, and lymphocytes. Fibroblasts, epidermal cells, endothelial cells, and other cells also contribute to tissue repair (Wynn, 2008). During the process of wound repair before tissue regeneration, these immune cells need to cooperate in an orderly manner. If there is toxicity or disorder in the process of tissue repair, it will not only delay tissue repair, but also result in excessive fibrosis or pathological scars, causing tissue regeneration to fail. Therefore, the repair process before tissue regeneration needs to be rigorously regulated (Mosser & Edwards, 2008).

Macrophages are widely distributed in tissues throughout the body and are key cells that induce the inflammatory immune response, including macrophages derived from the embryonic yolk sac and macrophages derived from bone marrow-derived monocytes (Martinez et al., 2008). The resident macrophages in human tissues are mainly derived from yolk sacs, fetal liver, and hematopoietic stem cells under normal physiological conditions (Zhang, 2018; Kishimoto et al., 2019). After injury, large numbers of monocytes reach the site of injury and resident macrophages present in the corresponding tissues or organs proliferate themselves in response to changes in the surrounding environment. Macrophages are extremely sensitive to the local microenvironment and respond to all forms of pathological injury by changing their complex phenotype and acquiring appropriate functions (Lee & Choi, 2018; Zhang, 2018; Louwe et al., 2021). When macrophages are polarized, they differentiate into M1 macrophages and M2 macrophages with opposite functions. M2 macrophages additionally are divided into four subtypes: M2a, M2b, M2c, and M2d. Each type has its own unique role (Wang et al., 2019). Macrophages are highly plastic and can differentiate into specific phenotypes under different stimulation. Macrophage depletion techniques have been widely used in a number of studies to investigate the relationship between macrophages and regeneration. The removal of macrophages at an early stage of tissue damage can directly lead to failure of tissue repair (van Rooijen & Sanders, 1997; Huen & Cantley, 2015), suggesting that macrophages are involved in regenerative activity to some extent.

Previous studies have mostly focused on the observation and description of the phenotype, morphology, and mechanism of macrophages that promote tissue damage by inducing an immune response (Rayees et al., 2020; Zhang et al., 2020a; Zhang et al., 2020b; Li et al., 2021a; Li et al., 2021b). With the gradual development of regenerative medicine and modern molecular biology, recent studies have found that macrophages play an important role in promoting the regeneration of different tissues such as the heart, liver, kidney, muscle, and nerve. Under different pathological microenvironments, macrophages regulate tissue regeneration by secreting regeneration-related cytokines, polarizing into different phenotypes, and even transdifferentiating into other cell types. The depletion of macrophages in different injury models can cause the regeneration of tissues and organs to be blocked. This review will focus on the important discoveries of macrophages in the field of regenerative medicine in recent years, and emphasize the indispensable role of macrophages in tissue repair and regeneration. The review will furthermore provide more insight for exploring tissue regeneration disorder caused by diseases with the goal of current research to develop new therapeutic strategies.

In past studies, researchers have concluded that macrophages are more involved in the regeneration process in some animal models with natural regeneration ability (such as zebrafish, salamanders, *etc.*), but not in non-amphibian vertebrates (Bevan et al., 2020; Simoes et al., 2020). The purpose of this review is to summarize the existing research on macrophages and tissue regeneration. To equip readers with a certain knowledge base, we begin with the nature of macrophages themselves. We conducted an extensive survey of published research on regeneration in non-amphibian vertebrates to delineate the specific roles and mechanisms of macrophages in different tissue types. It is worth noting that for some mammals that cannot achieve complete regeneration of tissues, accelerating tissue regeneration is feasible.

## Survey Methodology

To summarize the roles of macrophages in tissue injury and regeneration from multiple perspectives, the Web of Science and PubMed search engines were used to search the literature, and search terms included “cytokines,” “macrophage polarization,” “inflammation,” and “tissue regeneration”. In the process of summarizing the literature on tissue regeneration, we further refined the tissue classification. We searched the literature with two keywords for regeneration and macrophages, adding the tissue type (“heart,” “liver,” “kidney,” “nerve,” “muscle,” or “skin”).

## Macrophage-Derived Cytokines are Involved in Regeneration

In the complete cycle from injury to tissue regeneration, there are several phases, including the inflammatory response phase, the wound healing phase, and the tissue remodeling phase. A blastema formation phase is also present in specific animals, such as salamanders and zebrafish. The microenvironment in which the cells are located is varied at different times, and the interaction between cells relies on the response to their respective secreted cytokines. Some of the many cytokines released to the periphery by recruited macrophages are closely associated with tissue regeneration; they are highly expressed at some period during the regeneration process and act on other immune or non-immune cells in the environment, or act synergistically with other substances present in the environment to advance the progress of repair and regeneration.

### Pro-inflammatory cytokines

#### Tumor necrosis factor-*α* (TNF-*α*)

Tumor necrosis factor-*α* (TNF-*α*) is a soluble polypeptide chain composed of 157 amino acids (Carswell et al., 1975). As an important inflammatory mediator, TNF-*α* can stimulate and activate other immune cells and promote the infiltration of lymphocytes, neutrophils, and monocytes into the inflammatory site. In addition, TNF-*α* can coordinate the production of other cytokines, cell survival, and programmed cell death to ensure tissue homeostasis (Beutler, 1999). TNF- *α* is produced by activated macrophages and other types of cells such as CD4^+^T cells, neutrophils, and mast cells, and can induce inflammatory responses and apoptosis (Vieira et al., 2009; Griffin et al., 2012). Nguyen-Chi et al. (2018) found that TNF-*α* was released by macrophages in the early stage of zebrafish tail fin regeneration. TNF-*α* was determined to be one of the key signals transiently expressed by polarized macrophages in the early stage of regeneration. To control the regeneration process, the use of PTX (a non-selective phosphodiesterase inhibitor that can effectively inhibit the TNF-*α* reaction *in vitro* and *in vivo*) for treatment of zebrafish caudal fin will impair its regeneration process. After Nguyen-Chi et al. (2018) used morphine to inhibit the TNF-*α* receptor TNFR1, they found that the TNFR1/TNF-*α* axis is necessary for normal tissue regeneration. TNF-*α* promotes the recruitment of macrophages, which may be an enhanced cycle of macrophages accumulating to the wound area.

#### Interleukin-8 (IL-8)

IL-8, also known as CXCL8, belongs to the CXC subtype and is the first discovered cytokine with chemotaxis (Starckx et al., 2002; Gouwy et al., 2004). IL-8 is mainly secreted by monocytes and macrophages, and its function is to recruit and activate neutrophils for inflammatory response and cell killing. Therefore, IL-8 is also a strong inducer of neutrophils (Zeilhofer & Schorr, 2000). IL-8 acts by binding to its receptor. IL-8 has two receptors, CXCR1 and CXCR2. These two receptors are G protein-coupled receptors, and IL-8 can promote the development of inflammation by activating multiple downstream signaling pathways such as PI3K/Akt, PLC/PKC, and MAPK pathways after binding to the receptors (Russo et al., 2014; Zhang & Li, 2015; Sheikhpour, 2017). Tsai et al. found that after knocking down CXCL8 with morphine during the limb regeneration of axolotl, the regeneration rate of the limb was significantly reduced. At the same time, Tsai et al. found that after the undamaged limbs overexpressed IL-8, the recruitment of macrophages increased significantly. This research proved that CXCL8 plays an early immunomodulatory role during the limb regeneration of the axolotl, and through coupling with its homologous receptor CXCR-1/2, CXCL8 signaling is necessary for limb regeneration (de Oliveira et al., 2013).

#### Interleukin-6 (IL-6)

IL-6 is a key inflammatory cytokine. The relative molecular mass of IL-6 protein is 21 kda∼28 kda, and its specific size depends on the degree of glycosylation. It was first discovered as a B cell differentiation factor that stimulated B cell maturation and antibody production (Hunter & Jones, 2017). Monocytes/macrophages, T helper 2 (TH2) cells, vascular endothelial cells, and fibroblasts are the main producers of IL-6. IL-6 functions to induce inflammation and regulate immunity, and is involved in the development of inflammation. IL-6 not only regulates various immune functions, but also is the most potent cytokine for inducing acute phase proteins in the liver. Macrophages, endothelial cells, and smooth muscle cells produce a pleiotropic tumor necrosis factor that co-stimulates smooth muscle cells to produce IL-6 with interferon-*β* (IFN-*β*) and IL-1. IL-6 is a central regulator of inflammatory responses, stimulating the release of vasoactive substances, inducing fibrinogen secretion, and C-reactive protein production. IL-6R binds to the second membrane protein glycoprotein 130 (gp130), and gp130 dimerizes and initiates intracellular signal transduction. Macrophages quickly secrete IL-6 in the fight against infection symptoms and tissue damage, and promotes host defense by stimulating acute phase response, immune response, and hematopoiesis (Schaper & Rose-John, 2015; Garbers et al., 2018). Zhang et al. used knockout mice without the IL-6 gene and to study muscle injury models and found that IL-6 deficiency can seriously affect muscle regeneration; this is manifested as a significant decrease in the proliferation of myoblasts, an increase in interstitial fibrosis, and a smaller cross-sectional area of new muscle fibers. Lack of IL-6 can weaken the inflammatory response of damaged muscles, but an appropriate inflammatory response is necessary in the early stage of tissue regeneration (Zhang et al., 2013).

#### Interleukin-1 (IL-1)

IL-1 is mainly derived from macrophages and monocytes, but other types of cells such as epithelial cells, endothelial cells, and fibroblasts can also produce IL-1. IL-1 is the earliest discovered member of the IL-1 family and has three forms: IL-1 *α*, IL-1 *β*, and IL-1Ra. IL-1 *α* and IL-1 *β* exist in precursor and cleaved forms. Both forms of IL-1 *α* are biologically active, and only the cleaved form of IL-1 *β* is pyrogenic (Baker, Houston & Brint, 2019). IL-1 binds the IL-1 type 1 receptor, IL-1RI, with the same affinity, despite only 25% sequence identity between the first two forms. IL-1 *α* is a bifunctional cytokine, which is not only located in the nucleus to play a role in transcription, but also acts as a functional membrane-bound cytokine. When IL-1 *α* is expressed in the cytoplasmic matrix, IL-1 *α* enters the nucleus to mediate homeostatic functions, and is released after cell necrosis as an alarmin that induces inflammation (Voronov & Apte, 2015). IL-1 *β* is upregulated by inflammatory signals, promotes adaptive immune responses, and acts as an adjuvant for CD4^+^ and CD8^+^ T cell expansion and maturation (Ben-Sasson et al., 2009). IL-1 *α* and IL-1 *β* bind to IL-1R and recruit the IL-1R accessory protein and adaptor protein MyD88 to the receptor complex, leading to the activation of downstream signaling cascades that ultimately activate numerous immune and inflammatory genes. In order to inhibit the pro-inflammatory effects of IL-1 *α* and IL-1 *β* under appropriate circumstances, IL-1Ra is a naturally occurring anti-inflammatory molecule. IL-1Ra is classically secreted to competitively antagonizes IL-1 *α* and IL-1 *β*, and inhibits IL-1 *α* and IL-1 *β*, and IL-1 activity (Carmi et al., 2009).

Members of the IL-1 family are widely involved in various diseases, including cancer and liver failure, as well as liver regeneration. In recent years, more and more studies have reported that the IL-1 type I receptor signaling pathway affects liver regeneration to a certain extent, but its exact mechanism has yet to be determined. Early studies have shown that the IL-1R signaling pathway plays an important role in acute liver failure and liver regeneration after partial hepatectomy. Ma et al. (2016) found that IL-1 *β* siRNA adenovirus combined with MSCs reduced inflammation levels, prevented liver failure, promoted liver regeneration, and increased survival in mice with CCl4-induced acute liver failure, indicating an inhibitory effect of IL-1 *β* in post-acute liver regeneration liver failure.

#### Interleukin-12 (IL-12)

IL-12, also known as natural killer cell stimulating factor, is a heterodimeric cytokine composed of two subunits, p40 and p35, covalently bound by disulfide bonds. IL-12 is mainly produced by dendritic cells (DC), macrophages, B lymphocytes, and other antigen-presenting cells (APC), and is a growth-stimulating factor of immune cells with various biological effects. IL-12 can regulate the balance between Th1/Th2 cells and promote the proliferation of Th1-type cells (Dorman & Holland, 2000; Croxford, Kulig & Becher, 2014). IL-12 can also promote the differentiation and proliferation of T cells and NK cells, and induce production of interferon stimulating factor (GM-CSF) (Wang, Frank & Ritz, 2000). As a functional *γ* (IFN-*γ*), TNF- *α*, and granulocyte-macrophage colony bridge, IL-12 connects the early non-specific innate immunity and the later antigen-specific immune response, and plays an important role in the anti-infection process. However, as a pro-inflammatory factor, continuous secretion of IL-12 is not beneficial for tissue repair. Xu et al. found that IL-12 and IL-23 can delay bone formation and impair bone healing in their research on the p40 subunit of IL-12 for bone regeneration. However, the reduction of IL-12 and IL-23 can promote bone repair in the body. Since IL-12 and IL-23 both share the p40 subunit, the authors used a mouse model with total loss of IL-12 p40 and found that IL-12 p40^−/−^ mice stimulated bone formation and increased bone mass (Xu et al., 2016).

#### Anti-inflammatory cytokines

##### Interleukin-10 (IL-10)

IL-10 is a highly active multifunctional protein polypeptide molecule secreted by activated monocytes, T cells, B cells, and other cells (Mosser & Zhang, 2008). As a key anti-inflammatory cytokine, IL-10 has a wide range of anti-inflammatory effects *in vivo* and can be involved in all aspects of the inflammatory response. pro-inflammatory cytokines. IL-10 reduces the expression of MHC class II molecules on Its main biological role is to inhibit the inflammatory response and the release of the surface of dendritic cells and macrophages, weakens the function of antigen-presenting cells, and also inhibits the proliferation of T cells, which can effectively inhibit cell-mediated immune responses (Fiorentino et al., 1991; Chadban et al., 1998). IL-10 can also inhibit the production of IL-1 *β*, IL-6, IL-12, TNF, macrophage colony stimulating factor (M-CSF), and granulocyte colony stimulating factor (G-CSF). IL-10 is responsible for the production of factors such as platelet-activating factor (PAF) (Defrance et al., 1992; Rousset et al., 1992). IL-10 is a key molecule that antagonizes Th1 cells with a direct inhibitory effect, so IL-10 is defined as a cytokine synthesis inhibitor (Varzaneh et al., 2014). The secretion of IL-10 by cells depends mainly on the type of damaged tissue, the specific stimulus, and the timing of the immune response. Siqueira Mietto et al. (2015) found that after peripheral nerve injury in knockout mice without the IL-10 gene, the number of macrophages in the distal nerve segment increased significantly, indicating that IL-10 may play a role in controlling early infiltration and late outflow of nerves. In addition, IL-10 deficiency affected the phenotype of macrophages infiltrating the tissue after nerve injury, with CD206^+^ macrophages co-expressing CD86, and CD163^+^ macrophages co-expressing CD16/32 in IL-10^−/−^mice. This result suggests that IL-10 deficiency leads to a shift in macrophage phenotype towards a more pro-inflammatory phenotype. In terms of nerve regeneration, IL-10 deficiency negatively affects motor recovery, sensory recovery, and axonal regeneration of the sciatic nerve. Even in the late stages of nerve recovery, IL-10-deficient animals still have mechanically abnormal pain (Siqueira Mietto et al., 2015).

##### Transforming growth factor-*β* (TGF-*β*)

Transforming growth factor-*β* (TGF-*β*) is a cytokine first isolated from serum-free culture medium of mouse sarcoma virus-transformed embryonic fibroblast cell line in 1978, and it has stimulatory or inhibitory effects on a variety of cells (Becker et al., 2004; Travis & Sheppard, 2014). Human TGF-*β* mainly has three structurally similar isoforms: TGF-*β*1, TGF-*β*2, and TGF-*β*3. The three isoforms are highly homologous, but are encoded by different genes. Initially, the related genes form the precursor TGF-*β*, and then enter the Golgi apparatus for further processing and modification, and finally generate mature TGF-*β*. Under normal circumstances, the activity of TGF-*β* is inhibited. When the ionic strength changes in the body along with the formation of reactive oxidative substances (ROS), integrins and other substances, TGF- *β* signaling molecules are activated and released (Gleizes et al., 1997). TGF-*β* also plays a regulatory role in the repair and regeneration of the limbs of the axolotl. Tsai, Baselga-Garriga & Melton (2019) performed transcriptome sequencing after amputation of the axolotl limb and found that the growth factor signaling pathway involved in TGF-*β* is largely inhibited in regenerating tissues, and TGF-*β* only occurs in the early stages of regeneration (Tsai, Baselga-Garriga & Melton, 2019).

TGF-*β* is also a cytokine involved in skeletal muscle repair and regeneration. TGF-*β* is produced in skeletal muscle during injury response and plays a role in various stages of injury healing (Faler et al., 2006). During the inflammatory response phase, TGF-*β* can recruit more inflammatory cells and strengthen the inflammatory response; during the proliferative phase, TGF-*β* stimulates the production of extracellular matrix, angiogenesis, and epithelialization; and during maturation, TGF-*β* induces fibroblast generation and promotes wound healing by contraction. In addition, TGF-*β* can also regulate the proliferation and differentiation of satellite cells, which is specifically affected by the concentration of TGF-*β* (Faler et al., 2006). Allen & Boxhorn (1987) used TGF-*β* to culture mature rat skeletal muscle satellite cells *in vitro* and found that TGF-*β* could inhibit the proliferation and differentiation of satellite cells at the same time. The degree of differentiation of satellite cells is more sensitive to the effect of TGF-*β* concentration than its proliferation. A lower concentration of TGF-*β* can have an inhibitory effect on the differentiation of satellite cells (Allen & Boxhorn, 1987).

### Macrophage Phenotype Switching During Regeneration

Monocyte/macrophage lineage cells have two very significant characteristics: diversity and plasticity. In tissues, monocyte/macrophages acquire different functional phenotypes at any time in a variable environment, and they respond differently to microbial products, the activated lymphocytes, and damaged cells in the environment (Mantovani et al., 2002; Sica & Bronte, 2007; Biswas & Mantovani, 2010). In response to TLR ligand and IFN-*γ* or IL-4/IL-13 binding, macrophages undergo M1 (pro-inflammatory macrophages) or M2 (anti-inflammatory macrophages) activation (Geissmann et al., 2010). Once macrophages enter the tissue, they will acquire different morphological and functional properties under the guidance of the tissue microenvironment (*i.e.*, lung, bone, liver) and the other immune cells.

The successful regeneration of tissues requires macrophages to accurately switch their phenotypes. Any form of macrophages remaining in the tissue at an improper time or place will cause unavoidable negative effects.

### Pro-inflammatory macrophages exacerbate tissue damage

Continued recruitment of monocytes/macrophages or the inability to switch from a pro-inflammatory to a repairing phenotype at the right time can adversely affect wound healing and tissue repair. For example, in the process of peripheral nerve regeneration, M1 macrophages are mainly involved in the early Waller deformation, and M2 macrophages are mainly involved in the later nerve regeneration process. In addition, M1 macrophages have certain neurotoxicity, and the released reactive oxygen species, proteolytic enzymes, nitric oxide, etc. may kill neurons; in contrast, M2 macrophages are non-neurotoxic and have strong promoting axonal growth (Kigerl et al., 2009). Shimada et al.  used a chronic compression injury mouse model to measure hindlimb muscle mass and total bone mineral density. Macrophage infiltration was reduced in the macrophage-depleted group and in the drug-inhibited M1 macrophage group, and muscle mass and total bone density were significantly higher in the untreated group (Shimada et al., 2020). Therefore, M1 macrophage infiltration exacerbates muscle/bone atrophy after peripheral nerve injury, and muscle/bone atrophy can be avoided by inhibiting M1 macrophages localized to nerve injury.

Persistence of M1 macrophages leads to an ongoing inflammatory response that is lethal for successful tissue repair and regeneration. In the ischemia-reperfusion acute kidney injury model, the expression of M1-specific inflammatory factors (mainly IL-1 *β*, TNF-*α*, IL-12, IL-18, and IL-23) increased within 24 h after injury, and significantly decreased after three days (Lee et al., 2011). The activation of recruited macrophages begins to secrete these pro-inflammatory cytokines, which can lead to sustained enhancement of the inflammatory response at the injury site and acquisition of other inflammatory cells, such as Th cells. If M1 macrophages remain in their original shape after pathogenic or damaged cells are removed, their prolonged activation can lead to an excessive inflammatory response at the site of injury. Since the released cytotoxic molecules cannot distinguish between pathogens and normal cells, the cytotoxins will be detrimental to inflammatory absorption and damage repair. Excessive inflammatory response is also the main cause of many autoimmune kidneys damage (Kroner et al., 2014; Huen et al., 2015). Before ischemia-reperfusion injury, macrophages of all phenotypes induced by clodronate decreased, and transplantation of IFN-*γ*-activated macrophages after successful modeling resulted in aggravation of renal tubular injury (Lee et al., 2011).

Resident macrophages also undergo polarization under inflammatory stimuli. For example, in the acetaminophen-induced liver failure (AALF) model, Kupffer cells are polarized by inflammatory stimuli to generate M1 Kupffer cells, which release inflammatory mediators such as monocyte-derived macrophages and aggravate liver damage. A significant increase in the levels of IL-1*β* and TNF-*α* in the serum of AALF can be observed in the model, and the liver damage caused by acetaminophen can be significantly alleviated if the corresponding antibodies are used to inhibit these two factors (Antoniades et al., 2012). In conclusion, the dynamic changes in the number of M1 macrophages and M2 macrophages and the intensity of their effects play an important regulatory role in the occurrence, development, and prognosis of tissue damage. Effectively regulating the polarization direction of macrophages is an ideal strategy to deal with tissue or organ failure or necrosis and regeneration failure.

### The protective effect of anti-inflammatory and anti-fibrotic macrophages

Macrophages have more than a simple ability to promote the development of inflammation. Numerous studies have pointed to the mechanism by which macrophages exhibit anti-inflammatory and anti-fibrotic activity under altered tissue homeostasis. In a study on colitis, Shouval et al. (2014) showed that IL-10R signaling can delay colitis exacerbation, and this signaling process occurs in M2-type macrophages. On the contrary, if the expression of IL-10R receptor is defective, it will affect the polarization balance of monocyte-derived macrophages; that is, the ratio of pro-inflammatory macrophages to repairing macrophages is out of balance, leading to the exacerbation of colitis. Knuever et al. (2015) showed that the continuous production of pro-inflammatory factors by epidermal cells leads to excessive development of skin inflammation in mice, which is improved in mice lacking insulin and IGF-I receptors in bone marrow cells. In addition, the development and survival of Treg cells, which also produce IL-10 and TGF-*β*1, are regulated by repairing macrophages (Soroosh et al., 2013).

Hepatic macrophages can also eliminate apoptotic cells and reduce pro-inflammatory and pro-fibrotic signals depending on their phagocytosis. They also helps to terminate or reverse liver fibrosis while reducing liver inflammation. The deposition of the extracellular matrix (ECM) is currently thought to be a direct cause of liver fibrosis, in which different forms of matrix metalloproteinases (MMPs) play a key role in the degradation of fibrotic proteins, and anti-inflammatory Ly6C^low^ macrophages are the main source of MMPs (Pellicoro et al., 2012). The function of MMP-2, MMP-9, and MMP-13 are currently being researched more in the process of liver fibrosis regression, since they can encourage liver macrophages to degrade the ECM and reverse fibrotic lesions, while MMP-9 released by Kupffer cells can also cause hepatic stellate cell apoptosis (HSCs) (Baeck et al., 2012). CX3CR1 has also been shown to inhibit and reverse liver fibrosis by controlling the differentiation and survival of monocyte-derived macrophages. Ishida et al. (2017) reported that the ligand CX3CL1 of CX3CR1 not only induces the preferential expression of arginase I (ArgI) in wild-type mouse liver macrophages, but also binds to its receptor, promoting the survival of monocyte-derived macrophages through the Bcl-2 gene, thereby reducing the degree of liver fibrosis. In addition, tumor necrosis factor-related apoptosis-inducing ligand (TRAIL) synthesized and secreted by liver macrophages can induce the apoptosis of HSCs, reduce the expression of inhibitory metalloproteinases (TIMPs) in HSCs, and promote the degradation of extracellular matrix, thus playing a role in anti-fibrotic effect (Sun & Kisseleva, 2015).

## Macrophage Depletion Impairs Regeneration

Macrophages play a dual role in tissue injury and repair. Both monocyte-derived macrophages and tissue-resident macrophage populations contribute to the maintenance of normal tissue homeostasis and organ regeneration (Wynn, Chawla & Pollard, 2013). In a macrophage depletion study, Godwin, Pinto & Rosenthal (2013) used clodronate liposomes to deplete macrophages, after which they identified macrophages in injured adult salamander limbs necessary for successful regeneration. However, macrophages are also critical for clearing senescent cells (Yun, Davaapil & Brockes, 2015). The research team of Aurora et al. also used a similar cell depletion technique to deplete macrophages in mice. They found that in the absence of macrophages, newborn mice no longer had the ability to regenerate myocardium after myocardial infarction (Aurora et al., 2014).

Macrophage depletion generally refers to the removal of macrophages that have differentiated from circulating monocytes following tissue injury. Injection of clodronate liposomes or neutralizing antibodies to macrophage surface markers are the most commonly used methods. After the injection of clodronate liposomes, macrophages perceive it as a kind of substance similar to pathogens or microorganisms, so the macrophages will take it up and digest it. Clodronate will be released and accumulate in the macrophages. Once clodronates reach a certain concentration, apoptosis is induced in macrophages because of the toxicity. Macrophage depletion has a potential impact on the polarization of new monocyte-derived macrophages recruited after injury and determines regenerative outcome thereafter. A previous study on skeletal muscle regeneration has shown that different types of macrophages have different roles in muscle regeneration. M1 exacerbates muscle damage by producing pro-inflammatory cytokines and nitric oxide, while M2 promotes muscle repair, differentiation, and growth. Yet, macrophage depletion affects macrophage surface markers in damaged gastrocnemius muscle, with CD68 (M1 marker) remaining highly expressed during late regeneration and CD163 and CD206 (M2 marker) remaining low throughout the regeneration cycle, ultimately leading to accelerated fibrosis in damaged skeletal muscle (Xiao, Liu & Chen, 2016). In addition, macrophage depletion can also occur in specific organs. In the construction of a 2-acetylaminofluorene/partial hepatectomy rat model, depletion of macrophages using clodronate liposomes results in specific depletion of Kupffer cells in the liver (Xiang et al., 2012). The treated rats had increased mortality and increased liver damage, marked by decreased proliferation of hepatic progenitor cells (oval cells) due to macrophage depletion, with the most obvious consequence being a reduction in the regenerative capacity of residual liver tissue (Xiang et al., 2012). Reversible depletion of macrophages with AP20187 will severely impair functional recovery after neurological tendon injury in rats (Howell et al., 2021).

In the early stages of tissue regeneration, however, macrophages do not intervene much. The research from Wu et al. (2020) showed that macrophages have varying roles in different stages of recovery from acute pancreatic injury. The authors also used a similar macrophage depletion technique to eliminate macrophages before and after the occurrence of acinar ductal metaplasia (ADM) after pancreatic injury. The results indicated that depletion of macrophages before the formation of ADM, that is, at the beginning of pancreatic injury, not only leads to a decrease in macrophages, but also a reduction in the structure of ADM. Yet, the injured pancreas regenerates intact one week later. Depletion of macrophages during the formation of ADM will not only cause the ADM structure not to disappear, but also increase the number of immature macrophages. When the number of M2 macrophages is significantly reduced, repair functions cannot be performed and pancreatic tissue cannot be regenerated completely. Therefore, the period of existence and form of macrophages have a profound influence on each stage of the regeneration process (Aurora et al., 2014).

## Macrophages and Tissue Regeneration

### Macrophage and heart regeneration

Adult mammals do not have the same ability to fully regenerate hearts as newborns, and their heart injury is mostly manifested as fibrotic remodeling, but non-mammalian vertebrates and some newborn mammals have regeneration ability after heart injury. Numerous studies have found that zebrafish, some newborn mammals, and dragonflies have the ability to regenerate completely (Bevan et al., 2020). Exploring the relevant cells and mechanisms involved in the process of heart regeneration will help to provide a theoretical basis for the development of human heart regeneration capabilities (Lavine et al., 2014; Talman & Ruskoaho, 2016; Godwin et al., 2017).

**Figure 1:**
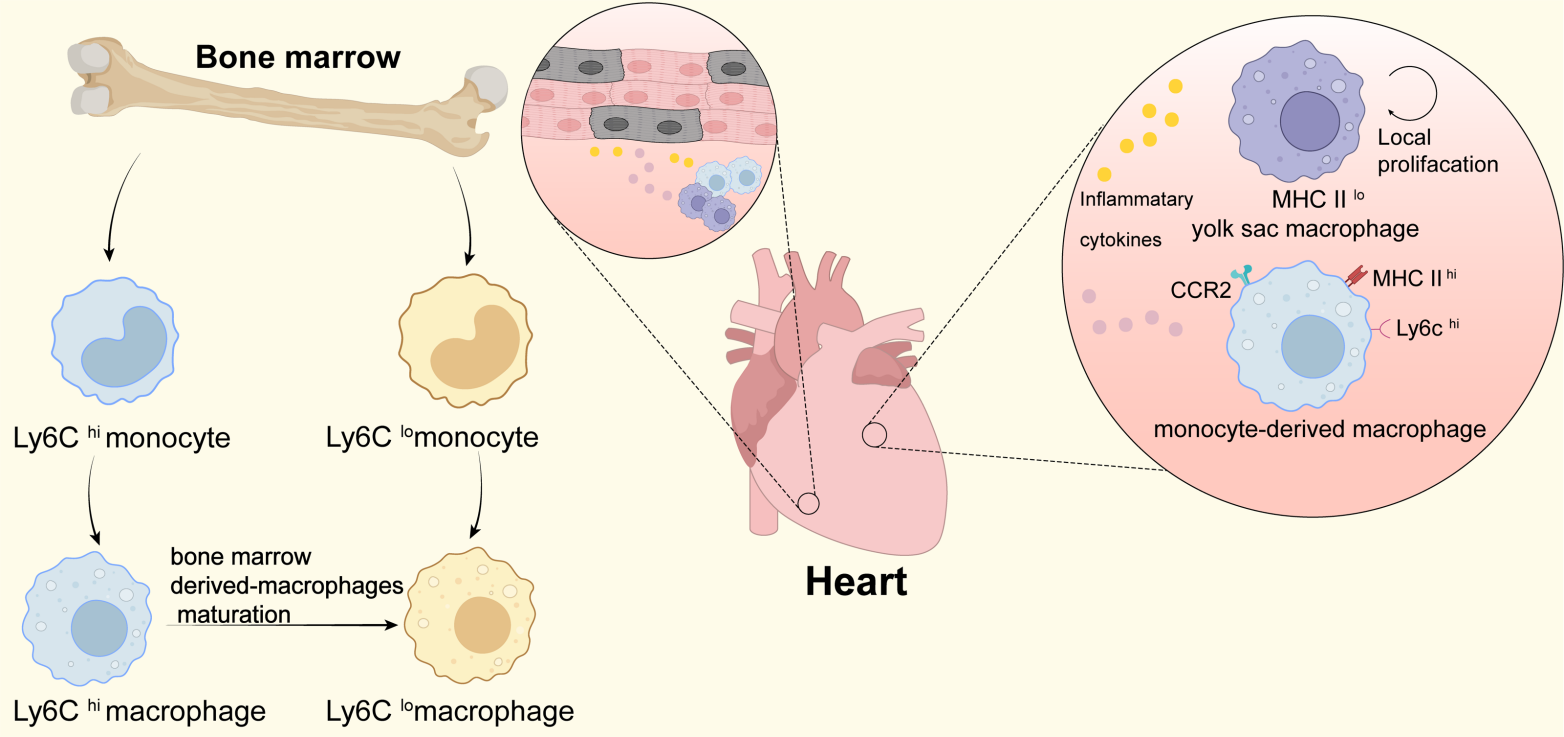
Macrophages in heart. This figure shows the origin of infiltrating monocytes in the bone marrow after tissue injury. Ly6C^hi^ macrophages can mature and differentiate into Ly6C^lo^ macrophages after infiltrating tissues. In the heart at homeostasis, tissue-resident macrophages are the predominant macrophage type, which originate from the yolk sac and are low in expression of MHC II (MHC II^lo^); during periods of stress in the heart, the number of cardiac macrophages increases significantly through local proliferation and monocyte recruitment. CCR2^+^ monocyte-derived macrophages and heart-resident macrophages produce and secrete large amounts of pro-inflammatory cytokines that promote cardiomyocyte regeneration and contribute to cardiac tissue remodeling.

Inflammation has been confirmed by many studies to help heart regeneration. Macrophages are a major cell type involved in the inflammatory response after myocardial infarction, which can regulate heart regeneration by triggering the inflammatory response. Macrophages in the damaged heart mainly include the resident macrophages derived from cardiac embryos and macrophages differentiated from peripheral monocytes. Researches have shown that after cardiac damage, newborn mice can selectively aggravate a small inflammatory response produced by cardiac resident macrophages (MHC-II^low^ CCR2^−^), inducing angiogenesis and cardiomyocyte proliferation, and promoting heart regeneration . Damaged hearts of adult mice mainly recruit MHC-II^high^ CCR2^+^ macrophages, which trigger a long-lasting inflammatory response and lead to cardiac fibrosis (Fig. 1). Further research found that monocyte chemoattractant protein-1 (MCP-1), MCP-3, chemokine (CXC motif) ligand 1 (CXCL1), leukocyte mediator Interleukin6 (IL-6), IL-1*β*, tumor necrosis factor- *α* (TNF-*α*) and other chemokines and inflammation-related factors have extremely low expression in MHC-II^low^ CCR2^−^ resident macrophages, and it is highly expressed in MHC-II^high^ CCR2^+^ macrophages. Targeted fine-tuning of macrophage related cytokine expression may contribute to the regeneration of injured hearts. In addition, the use of CC chemokine receptor 2 (CCR2) selective inhibitors prevents the recruitment of monocyte-derived CCR2^+^ macrophages in the damaged heart of adult mice, while retaining CCR2^−^ resident macrophages. Using CCR2 showed that inflammation in the damaged area of the heart was reduced, and the density of coronary micro-angiogenesis increased (Lavine et al., 2014). The results suggest that CCR2 inhibitors are expected to be used in the targeted therapy of clinical myocardial infarction. Interestingly, another research found that overexpression of CCL2 (the ligand of CCR2) in the heart induces massive infiltration of CCR2^+^ monocytes and macrophages, which can promote cardiac angiogenesis and improve left ventricular dilatation and dysfunction after myocardial infarction (Lavine et al., 2014). Thus, CCR2^+^ macrophages may play a dual role in the repair of heart damage (Lavine et al., 2014).

Macrophages can also promote heart regeneration by regulating the production of the extracellular matrix (ECM). The ECM can provide a structural framework for cardiomyocytes and participate in biochemical and electrical signal transmission (Talman & Ruskoaho, 2016). Researchers have found that zebrafish and salamander ventricles form a special ECM collagen network after freezing damage. The network is to some extent helpful for the heart to return to normal function, which allows activated proliferating cardiomyocytes to migrate and participate in the repair of damaged myocardial walls (Hortells, Johansen & Yutzey, 2019). When macrophages are depleted, the number of fibroblasts in the damaged heart is significantly reduced. Additionally, the characteristics of ECM synthesis, remodeling, and cross-linking are significantly altered. Highly cross-linked fibrotic scars are formed in the damaged area, therefore interrupting heart regeneration. The latest research has found that macrophages can also autonomously deposit collagen through transdifferentiation into myofibroblast-like cells, and promote the regeneration of damaged zebrafish hearts (Simoes et al., 2020). In addition, a RNA-Seq gene transcription analysis of macrophages after heart damage at different developmental stages in mice determined that after myocardial injury in newborn and adult mice, macrophages regulate the expression of ECM-related genes to achieve regeneration repair transition to fibrosis repair (Simoes et al., 2020). The results confirm that macrophages can directly or indirectly regulate ECM to promote heart regeneration. It is worth noting that in the death of myocardial cells caused by ischemia/reperfusion injury in humans, the rapid deposition of early ECM helps prevent heart rupture, but excessive deposition of ECM can contrastly cause cardiac fibrosis and heart failure. Further investigating the differences of macrophages regulating ECM to promote cardiac regeneration or cardiac fibrosis will help provide new therapeutic ideas for cardiac regeneration therapy.

### Macrophages and liver regeneration

The liver has a high degree of regeneration ability after being partially removed or damaged by toxins (Izumi et al., 2018). During liver regeneration, the remaining parenchymal cells and non-parenchymal cells proliferate and replace lost tissues through complex interactions, thereby achieving liver regeneration (Zafarnia et al., 2019). Macrophages derived from Kupffer cells and monocytes account for about 20% of total liver cells and play an important role in liver regeneration (Krenkel & Tacke, 2017; Shan & Ju, 2020).

**Figure 2:**
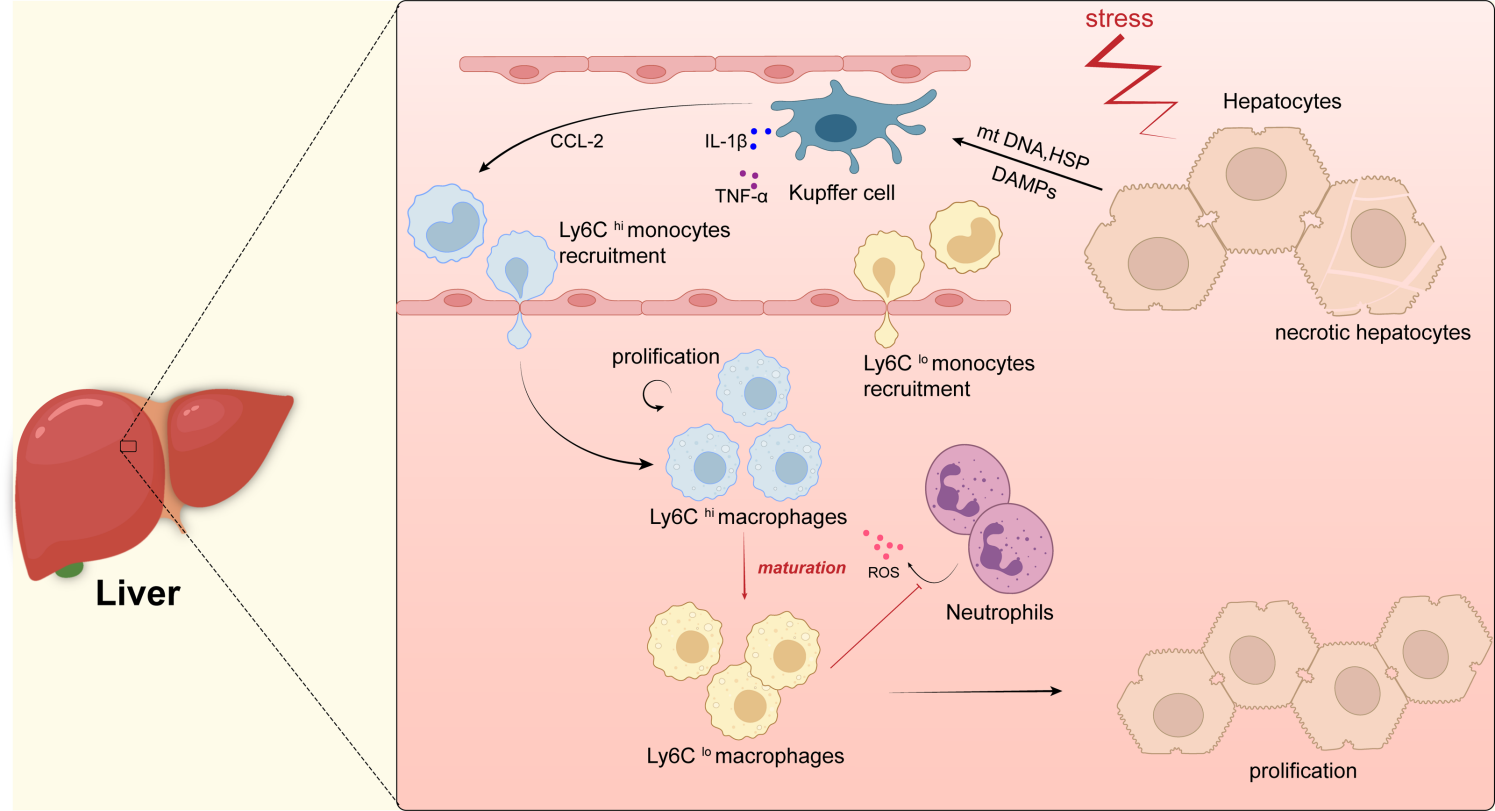
Macrophages in liver. Kupffer cells are immobilized within the lumen of liver sinusoidal endothelial cells (LSECs) and when hepatocytes are damaged, intracellular components, such as mitochondrial DNA (mtDNA) or heat shock proteins (HSP), are released as dangerously relevant molecular patterns (DAMP). DAMPs activate Kupffer cells, which in turn secrete inflammatory cytokines (*e.g.*, TNF- *α*, IL-1 *β*, etc.) as well as CCL-2. CCL-2 recruits Ly6C^hi^ monocytes from the blood to infiltrate tissues and differentiate into pro-inflammatory macrophages (Ly6C^hi^), which will mature into Ly6C^lo^ macrophages with restorative and anti-inflammatory properties when inflammation resolves, they promote the regeneration of liver parenchymal cells and thus the repair of the liver. In addition, Ly6C^lo^ macrophages inhibit the secretion of reactive oxygen species (ROS) by neutrophils.

Kupffer cells are tissue-specific macrophages residing in the liver and are located in the sinusoidal cavity around the liver cells. As a key role in the process of liver regeneration, Kupffer cells can induce the proliferation of hepatocytes through paracrine growth regulators. Studies have shown that Kupffer cells reach a peak of proliferation 72 h after partial liver resection, itBy then, Kupffer cells promote liver parenchymal regeneration by up-regulating the expression of nuclear factor- *κ*B(NF-*κ*B) to moderate the cell cycle and proliferation of cytokines TNF-*α* and IL-6 (Shan & Ju, 2020). After partial hepatectomy involving the depletion of Kupffer cells, the activity of NF-*κ*B in the liver tissue was lost, the concentration of TNF-*α* and IL-6 in the serum decreased, and the proliferation markers PCNA and cell cycle specific protein cyclinB1 were downregulatied, all of which leads to delayed cell proliferation and damages liver regeneration (Shwartz, Goessling & Yin, 2019). Macrophages of monocytes origin are another key role in the process of liver regeneration. Graubardt et al. (2017) showed that Ly6C^low^ macrophages differentiated from Ly6C^high^ monocytes accelerated the regression of inflammation and promoted liver regeneration in paracetamol-induced drug-induced liver injury by reducing infiltration of ROS-producing neutrophils. In the absence of Ly6C^low^ macrophages, neutrophils accumulate in large numbers and produce ROS, triggering a severe inflammatory response that can lead to liver injury. Moreover, Graubardt et al. found that selective ablation of Ly6C^high^ monocytes and Ly6C^low^ macrophages impair the regeneration of the liver (Fig. 2).

In addition, recent research on drug-induced acute liver injury found that using CCL5 inhibitors or CCL5 neutralizing antibodies to induce macrophages to polarize from the M1 to M2 phenotype can promote liver parenchymal regeneration, thereby significantly reducing paracetamol-induced acute liver injury (Li et al., 2020). The results indicate that CCL5 inhibitors and CCL5 neutralizing antibodies are expected to be used in the clinical treatment of drug-induced liver injury. Meanwhile, investigation of the mechanism that promotes the polarization of macrophages to the M2 phenotype after liver injury may provide a new strategy for the treatment of acute liver injury. Another research on chronic liver injury found that after hepatocyte death, macrophages can engulf necrotic cell fragments to up-regulate the expression of Wnt3a gene, and further activate the classic Wnt signal in hepatic progenitor cells (HPC) to induce HPC. Differentiation of hepatocytes promotes the regeneration of liver parenchyma in chronic liver injury (Boulter et al., 2012). In addition, the research also found that macrophages can up-regulate Numb expression through the Wnt3a signaling pathway, thereby inhibiting Notch signaling and blocking the differentiation of HPC into bile duct cells. The interaction between the different signaling pathways involved in macrophages is essential for the correct regulation of liver regeneration.

In summary, different types of macrophages play an important role in the regeneration of the liver after injury. Promoting the polarization of macrophages to the M2 phenotype or selectively inducing Wnt3a expression provides a new therapeutic strategy for the treatment of liver injury. The current research on the promotion of liver regeneration by macrophages is mainly focused on monocyte-derived macrophages or Kupffer cells. However, due to the highly heterogeneous characteristics of macrophages, whether there are other cells transdifferentiating into macrophages that contribute to liver regeneration has not been reported in the literature.

### Macrophages and kidney regeneration

Kidney cells have a certain ability to recover after ischemia/reperfusion injury or toxic injury, depending on the regeneration of renal tubular epithelial cells according to the nature and location of the injury (Urbschat et al., 2020). Macrophages are a main cell type that infiltrate damaged kidneys and play an important role in kidney regeneration (Urbschat et al., 2020). Clarifying the relevant mechanisms of macrophages regulating kidney regeneration will help provide new therapeutic methods for the treatment of acute and chronic kidney injury.

**Figure 3:**
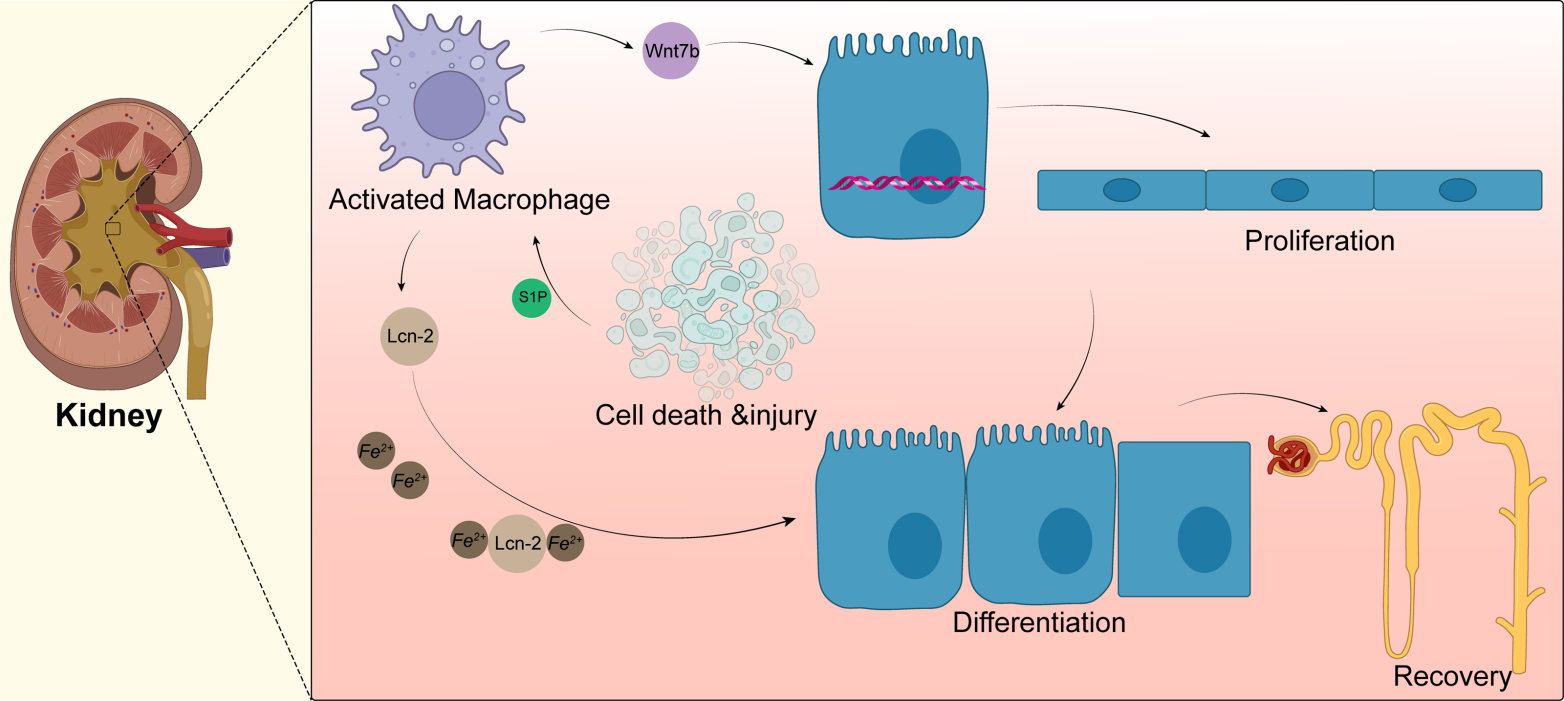
Macrophages in kidney. Acute kidney injury can lead to tubular epithelial cell death and injury, resulting in macrophage recruitment and activation. Activated macrophages promote the activation of the Wnt pathway, as shown by the upregulation of Wnt7b in macrophages, which directs the proliferation of renal tubular epithelial cells; macrophages also secrete lipid transport protein 2(LCN-2), and apoptotic cells secrete sphingosine-1-phosphate to promote the secretion of more Lcn-2 by macrophages, which binds to iron ions for transport to epithelial cells and promotes epithelial cell differentiation.

Macrophages can drive kidney regeneration by triggering Wnt signals. After kidney injury, the Wnt ligand Wnt7b in macrophages is significantly upregulated. The G2 cell cycle stagnation of renal epithelial cells can be solved by activation of the Wnt pathway, which can also guide cell progression, basal membrane repair, and renal tube regeneration. After the ablation of macrophages, the level of molecular activation of Wnt pathway-related signals in renal epithelial cells decreased; the same phenomenon is shown in kidney repair and regeneration. When Wnt7b is missing in macrophages, the normal regeneration of renal tubular epithelial cells is blocked. Thus, the plasma creatinine value cannot return to normal levels, and the kidney repair ability is weakened. Yet, the kidney repair ability is significantly enhanced after injection of the Wnt pathway agonist Dkk2 (Huffstater, Merryman & Gewin, 2020). At present, the specific mechanism of Wnt7b^+^ macrophages regulating kidney regeneration and whether these macrophages participate in regulating the regeneration of other tissues and organs remains to be further studied. Targeting up-regulation of Wnt7b in macrophages may provide a new treatment model for promoting the repair of human tissues and organs (Fig. 3).

Macrophages can also promote the regeneration of renal tubular epithelial cells after kidney injury in mice by secreting lipocalin-2 (Lcn-2) (Weigert, Olesch & Brune, 2019). After renal tubular injury, sphingosine-1-phosphate (S1P) secreted by apoptotic cells promotes the production and secretion of large amounts of Lcn-2 by macrophages, which can bind and transport iron to epithelial cells, promote the proliferation of renal epithelial cells, and induce the regeneration of renal tubules. Injection of macrophages overexpressing Lcn-2 through the tail vein of mice promotes the proliferation of renal tubular epithelial cells, and this process can be blocked by Lcn-2 neutralizing antibodies (Jung et al., 2016). Rota et al. (2018) found that human bone marrow mesenchymal stem cells mediate the polarization of macrophages to the M2 anti-inflammatory phenotype by homing to the kidney, which further promotes the regeneration of damaged kidneys caused by ischemic/re-perfusion. In renal stone disease, M2 macrophages can inhibit renal crystal formation. Calcium oxalate stones are the most common type of disease and are closely associated with the macrophage phenotype. Sirtuin3 overexpression significantly increases the expression of M2-related genes (Arg-1 and IL-10) and inhibits renal inflammation, renal apoptosis, and calcium oxalate crystal deposition. Sirtuin3 promotes FOXO1 deacetylation, thereby indirectly promoting macrophage polarization towards M2 (Xi et al., 2019). However, recent studies have also shown that the polarization of macrophages towards M2 is not always positive. Some disease models, such as the aristolochic acid-induced nephropathy model performed by Wang et al. (2022) showed that persistent M2 infiltration promotes renal fibrosis and prevents the recovery or regeneration of pathologically damaged renal tissue. MicroRNA-382 is an endogenous non-coding small RNA that significantly suppresses CD206^+^M2 numbers after aristolochic acid stimulation following knockdown or knockout of microRNA-382. In addition, Wang et al. found an interaction between macrophages and renal tubular cells in a co-culture system, with downregulation of apoptosis-associated and epithelial mesenchymal transition (EMT)-associated proteins following microRNA-382 knockdown. Conversely, overexpressing microRNA-382 in macrophages will promote apoptosis and EMT in renal tubular epithelial cells (Wang et al., 2022). Members of the signal transducer and activator of transcription protein family have recently been found to have some association with chronic kidney disease and recovery, and this association may be negative. Pharmacological inhibition or knockdown of STAT6 prevents macrophages from polarizing towards M2. Since M2 mediates the conversion of monocytes to bone marrow-derived fibroblasts, inhibition of STAT6 can improve renal fibrosis and promote the restoration of normal kidney function. This finding was confirmed in both the unilateral ureteral obstruction nephropathy model and in folic acid nephropathy (Jiao et al., 2021a; Jiao et al., 2021b). Similarly, targeted inhibition of STAT3 resulted in a significant reduction in collagen deposition in renal interstitial fibrosis, as evidenced by reduced expression of alpha smooth muscle actin (*α*-SMA), collagen I, fibronectin, and vimentin (Wang et al., 2022).

In short, macrophages can play an important role in kidney regeneration through different pathways. At present, there are few reports on the regulation of renal regeneration by macrophages. Further research on the mechanism of macrophages-induced renal regeneration may be inspiring for the treatment of acute and chronic kidney injury and related complications.

### Macrophages and peripheral nerve regeneration

**Figure 4:**
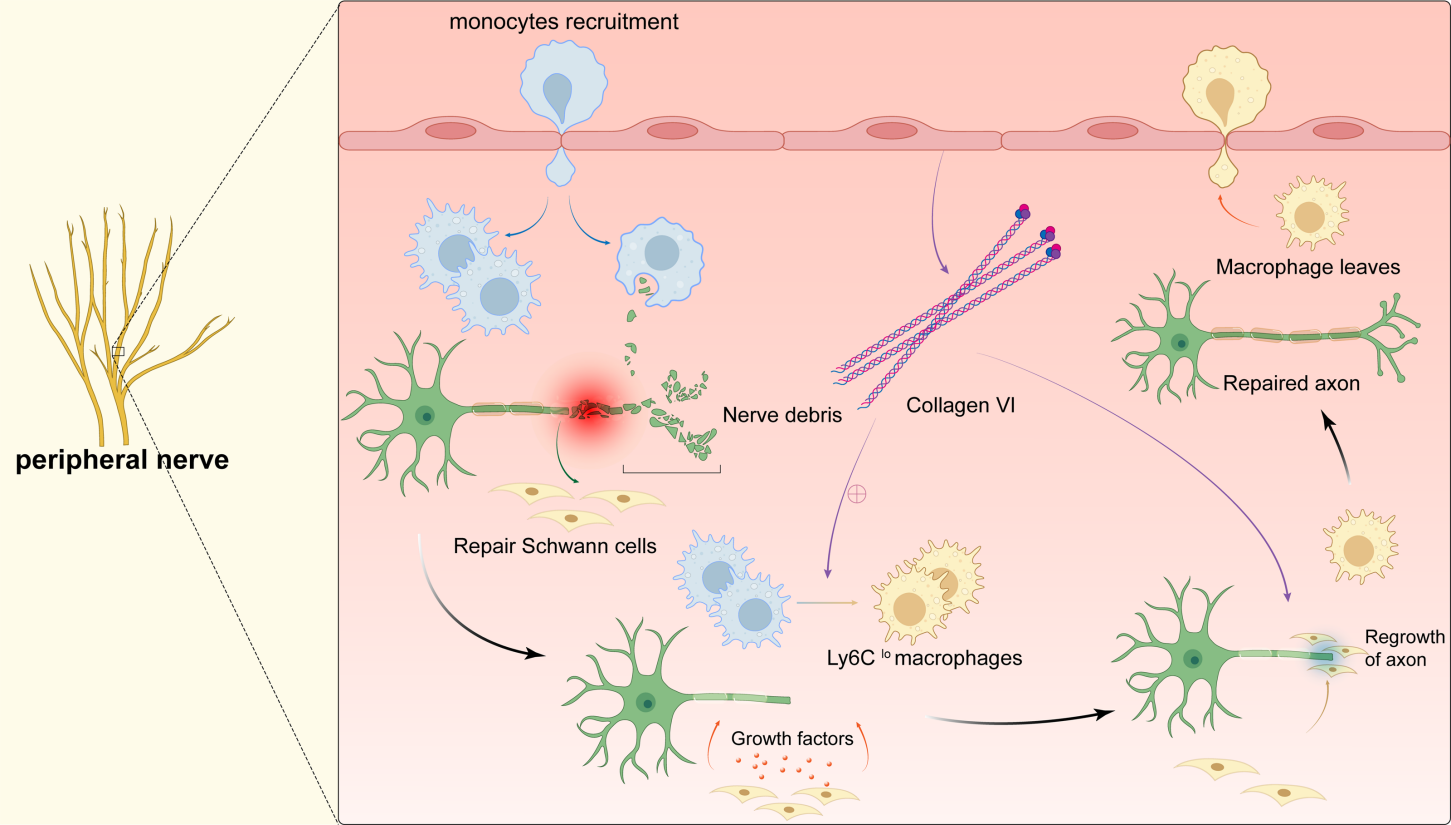
Macrophages in peripheral nerve. After peripheral nerve injury, monocytes are recruited to the site of injury and differentiate into pro-inflammatory macrophages, macrophages phagocytose myelin debris, and Schwann cells isolate and dedifferentiate into repairing Schwann cells, which secrete cytokines to promote axonal regeneration while the pro-inflammatory macrophages mature into repairing macrophages (Ly6C^lo^). The polarization of macrophages stimulates the secretion of collagen VI from the membranes of the peripheral nerve, which induces macrophage polarization by positive feedback and participates in axonal repair. The repairing Schwann cells guide axonal regeneration and the repairing macrophages release anti-inflammatory cytokines. After complete nerve regeneration, the macrophages withdraw from the peripheral nerve tissue.

Once the peripheral nervous system (PNS) is damaged, axon regeneration and remynelgation capacity can be adversely affected or even lost (Peluffo et al., 2015; Li et al., 2019). Therefore, suture reconstruction technology is currently used clinically to promote the repair of damaged peripheral nerves. However, because the sutures cannot completely seal the nerves, important substances leak out, and the operator has extremely high technical requirements. As a result, fibrotic scars are formed on the damaged parts, which are almost impossible to recover again (Liu et al., 2019a; Liu et al., 2019b). Exploring the relevant mechanisms involved in the process of peripheral nerve regeneration will help to develop new regenerative therapies for nerve injury. After bestowing transverse damage to the peripheral nerve, the distal axon is separated from the cell body. The distal axon degenerates rapidly through Waller degeneration. Subsequently, the myelin ovum disintegrates to produce a large number of myelin fragments, and the expression of regeneration-related genes in the neuron cell body is up-regulated to promote the regeneration of the proximal axon, and finally remyelination to complete the nerve regeneration (Fig. 4). During this process, delayed Waller degeneration and slow or insufficient removal of myelin necrotic fragments can inhibit axon regeneration (Stoll, Jander & Myers, 2002). Complete peripheral nerve regeneration requires the macrophage involvement. Macrophages can promote axon regeneration and remyelination by removing fragments of the myelin sheath, secreting growth factors, and inducing angiogenesis (Chen, Piao & Bonaldo, 2015; Liu et al., 2019a; Liu et al., 2019b; Matsui et al., 2022. Clarify the relevant mechanism of macrophages in promoting peripheral nerve regeneration can provide a new treatment strategy for targeted macrophages to treat damaged nerves.

When peripheral nerve damage occurs, a large number of macrophages are recruited to the injured area to promote peripheral nerve regeneration through a variety of ways. Forese et al. (2020) found that L-prostaglandin D2 synthase (L-PGDS) can enable macrophages to acquire appropriate phagocytic capacity to remove myelin debris through a non-cell-autonomous mechanism, which is conducive to axon regeneration and remyelination. At the same time, macrophages can also remove debris by regulating the phagocytic ability of Schwann cells and promote axon regeneration. Recently, researches have shown that macrophages can promote peripheral nerve regeneration by inducing the production of new blood vessels. In the process of regeneration of injured peripheral nerves, the two nerve stumps must be reconnected with a nerve bridge tissue, and then collectively migrated by Schwann cells to guide the regenerated axons to cross the bridge, ensuring normal regeneration of PNS. Macrophages secrete vascular endothelial growth factor-A (VEGF-A) in response to hypoxic environment, which relieves hypoxia by inducing a specific polarized new blood vessel in the bridge area. Schwann cells collectively migrate under the guidance of the blood vessel and mediate regenerated axons to bridge. Disrupting the neovascularization induced by macrophages will cause the cells to misorientate and migrate into the surrounding tissues, which results in regenerating axons fail to bridge and ultimately impairing normal nerve repair (Qun, Xinjie & Hui, 2018). In addition, macrophages regulate peripheral nerve regeneration by polarizing to the M2 phenotype to promote the secretion of type VI collagen. Collagen VI acts through positive feedback regulation to further promote macrophages to M2-type switching, in order to achieve nerve regeneration success. Studies in animal models found that, the assembly and secretion of collagen VI in Col6a1^−/−^ mice were blocked compared with control mice, which causes M2 polarization to be blocked and regeneration delayed after transplantation of wild type bone marrow cells. This reestablishs the existence of peripheral nerves (Lv et al., 2017). Another study found that oxidized galectin 1 can promote the secretion of axon growth factors and Schwann cell migration factors from macrophages, and promote axon regeneration and Schwann cell migration (Shen et al., 2018). Promoting the recruitment of macrophages and timely polarization to M2, enhancing the phagocytic potential of macrophages, and correctly inducing polarization vascularization may provide new therapeutic ideas for the treatment of peripheral nerve damage-related diseases.

### Macrophages and skeletal muscle regeneration

Proliferation and differentiation of satellite cells give skeletal muscle remarkable regenerative capacity (Patsalos et al., 2019). Part of the activated satellite cells are transformed into myoblasts, which form myotubes through migration and fusion contact with each other. The newly formed myotubes grow and transform into mature muscle fibers to achieve skeletal muscle regeneration. Macrophages are involved in triggering the inflammatory immune response after skeletal muscle injury. They can promote skeletal muscle regeneration through various ways such as activating satellite cells and promoting angiogenesis (Patsalos et al., 2019).

**Figure 5:**
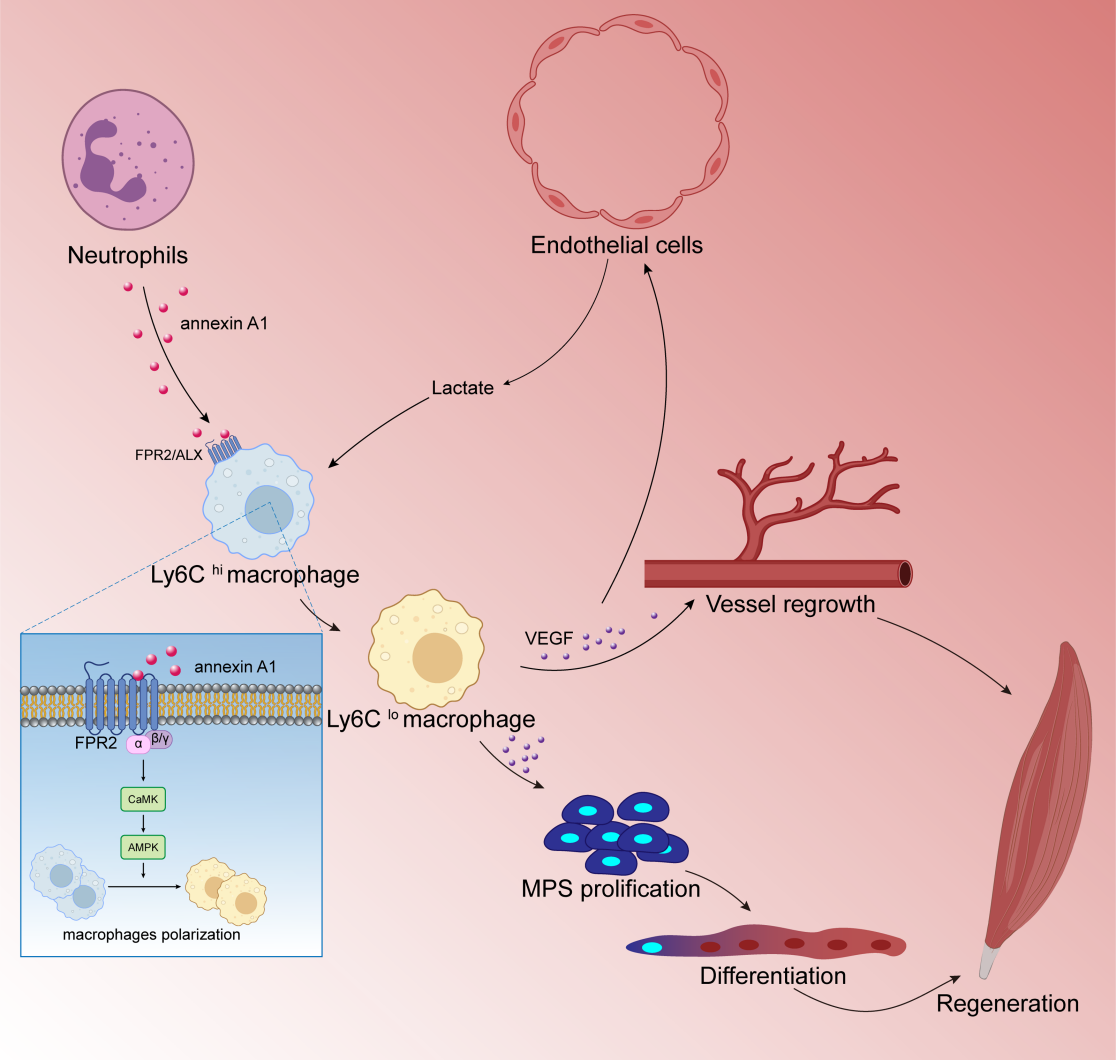
Macrophages are involved in skeletal muscle regeneration. Skeletal muscle is a highly vascularized tissue and endothelial cells are the main cell type of blood vessels. During muscle regeneration, endothelial cells release lactic acid, which directly controls macrophage function. Under lactic acid stimulation, pro-inflammatory macrophages differentiate into reparative macrophages, which secrete large amounts of vascular endothelial growth factor (VEGF), actively supporting muscle vascularization and forming a positive feedback pathway. VEGF also induces the proliferation and differentiation of myogenic progenitor cells (MPCs) in skeletal muscle. Muscle regeneration depends on the self-renewal of MPCs to replenish the muscle stem cell pool, and MPCs repair damaged muscle by differentiating and fusing with each other; following injury to skeletal muscle, membrane linked protein A1 (Annexin A1), which is secreted in large numbers by neutrophils, activates the FPR2/ALX receptor on the surface of macrophages and the downstream adenylate-activated protein kinase signaling (AMPK) pathway, promoting the conversion of pro-inflammatory macrophages to reparative macrophages.

Numerous studies have shown that M2 type anti-inflammatory macrophages can promote skeletal muscle regeneration through various methods. Jiang et al. (2020) found that cannabinoid receptor subtype 2 (CB2R) mediates the polarization of macrophages to the M2 phenotype, which is beneficial to mouse skeletal muscle regeneration. After knocking out CB2R, the following ensues: M2 type polarization is blocked; the expression of myogenic regulators MyoD and myogenin is down-regulated; and satellite cell activation is reduced. These reactions lead to a significant decrease in the number of new central nuclear myotubes and in the volume of regenerated muscle fibers, leading to necrosis sexual muscle fibers while inflammatory cell infiltration increased significantly. Research by Zhang et al. (2020a); Zhang et al. (2020b) showed that endothelial cells initiate lactate shuttle through glycolysis products during ischemia, which polarizes macrophages to M2 type and secretes a large amount of VEGF, which strengthens the positive feedback loop of angiogenesis, improves the density of angiogenesis, and promotes muscle revascularization. In addition, VEGF can also contribute to the muscle regeneration by inducing the proliferation and differentiation of myogenic progenitor cells of skeletal muscle. McArthur et al. (2020) showed that after skeletal muscle injury, macrophages activate FPR2/ALX receptors and downstream adenylate-activated protein kinase (AMPK) signals by up-regulating annexin A1 (annexin A1) expression to promote the polarization of macrophages to M2 type and inhibit the inflammatory response.Thereby, muscle regeneration can proceed successfully (Fig. 5). Iavarone et al. (2020) found that Cripto expression in CD206 (M2 marker) positive macrophages after skeletal muscle injury was up-regulated and partially inhibited endothelial-mesenchymal transition (EndMT) to promote vascular remodeling, which enhances muscle regeneration potential. After ablation of the Cripto gene of myeloid cells, the number of CD206 positive cells was significantly reduced, but the degree of EndMT increased. This in turn interfered with vascular remodeling and significantly decreased muscle regeneration. In addition, studies have found that mesenchymal stem cells and extracellular matrix can also promote muscle regeneration by regulating the polarization of macrophages to the M2 type (Qiu et al., 2018).

The above research results provide a sufficient theoretical basis for M2 type macrophages to promote skeletal muscle regeneration. Reasonably regulating the polarization of macrophages to M2 type may bring higher clinical benefits for muscle regeneration therapy. In addition, some studies have shown that after skeletal muscle injury, macrophages can activate receptor *γ* through increased expression of peroxisome proliferators (PPAR *γ*) (Dort et al., 2019), mechano growth factor (MGF) (Liu et al., 2019a; Liu et al., 2019b), adisintegrin-like and metalloproteinase with thrombospondin type 1 motif (ADAMST1) (Du et al., 2017), and insulin-like growth factor-11 (IGF-11) (Zhang et al., 2019) to promote skeletal muscle regeneration.

### Macrophages and skin regeneration

The skin of organisms undergoes a dynamic and highly controlled recovery process after injury, and the different types of cells and intra- and intercellular signaling pathways involved in this process have been extensively studied (Gurtner et al., 2008). This process consists of four successive, intertwined phases: the blood clotting phase, the inflammatory phase, the proliferative phase, and the tissue remodeling phase, which represent the principles of repair in most tissues, with monocytes/macrophages involved in almost the entire process of wound healing. Resident macrophages are also to be found in the skin, known as epidermal Langerhans cells (LC), dermal resident macrophages, and dendritic cells (DC). Langerhans cells in the skin are mainly associated by dendritic protrusions and are distributed in a grid-like pattern in the epidermal spine layer, accounting for 3%–5% of the total epidermal cells (Merad, Ginhoux & Collin, 2008). When the skin is injured and an inflammatory response occurs, LC gradually migrates to the draining lymph nodes of the skin and plays a role in presenting antigens to helper T lymphocytes in the lymph nodes, eventually differentiating into mature LC (Doebel, Voisin & Nagao, 2017). Under normal conditions, LCs, dermal DCs, and macrophages renew themselves through slow self-proliferation. When skin tissue is damaged, these cells are mainly replenished by monocytes in the peripheral blood (Sere et al., 2012; Kolter et al., 2019).

As early as 1978, Greenburg and Hunt recognized that infusion of activated *macrophages in vitro* could regulate wound angiogenesis and thus promote wound healing (Greenburg & Hunt, 1978). In the early stages of wound healing, M2 macrophages secrete vascular growth factors such as VEGF, IGF-1, TGF *β*, TNF-*α*, IL-6, IL-8, and IL-10 to chemotactic endothelial cells to reach the wound and promote their proliferation and differentiation to form tube-like structures. Matrix metalloproteinases (MMP), elastases, and serine proteases released by macrophages degrade the ECM, which facilitates the migration of endothelial cells and vascular smooth muscle cells to the site of angiogenesis and the reformation of vessel wall cells (Wynn & Vannella, 2016). In later stages of healing, macrophages also secrete platelet responsive protein (TSP-1) to promote the regression of new capillaries and prevent excessive vascular proliferation (Agah et al., 2002).

During the tissue remodeling phase, inadequate repair will lead to excessive matrix degradation, resulting in delayed healing or even chronic wounds, while excessive repair will lead to massive collagen deposition and pathological hypertrophic scarring (Finnerty et al., 2016; Morton & Phillips, 2016). The scar tissue continues to reconstruct and regain some functions, but the composition still very different from normal skin tissue. Unlike the typical acute wound healing process, the conversion of M1 and M2 macrophages is dysregulated in chronic wounds, with 80% of the wound edges being M1 macrophages, which is the biggest contributor to persistent inflammation and eventual scarring of chronic wounds (Oishi & Manabe, 2018). A study using single-cell sequencing of salamander limb regeneration revealed the presence of M2 macrophages early in the regeneration process, suggesting that the specific immune microenvironment constituted by the presence of M2 subtypes early in the limb regeneration process is likely to be the key to scar-free regeneration in salamanders (Li et al., 2021a; Li et al., 2021b). Of course, it is not possible to absolutely polarize macrophages towards a particular subtype, as this would not only not trigger regeneration, but might make even the most basic wound healing difficult. A study using BALB/c recombination-activated gene (Rag)-2 and IL-2 receptor *γ* (IL-2R *γ*) double knockout (KO) mice to study wound healing found that M1 hyperpolarization, poor angiogenesis, and trauma damage were exacerbated by congenital loss of regulatory function in KO mice (Seraphim et al., 2020).

## Conclusion

This review summarizes recent studies on the numerous roles played by macrophages during the regeneration of different tissues in mammals, with reference to past studies on amphibian vertebrates with a gift for regeneration. After comparing studies, it was found that the ability to achieve scar-free repair after severe trauma to organs in adult life is the biggest difference between mammals and these amphibian vertebrates. Macrophages are immune cells that are involved in nearly the entire process of tissue regeneration, relying on their versatile morphology. M1 macrophages and M2 macrophages are the two most studied phenotypes, and these phenotypes shift flexibly at different stages of tissue regeneration. This review emphasizes that it is not absolute whether any form of macrophage is beneficial or disadvantageous for tissue regeneration, as macrophages play distinct roles in specific processes in different tissues. At present, studies on macrophages during mammalian tissue regeneration still lack understanding of some ideal model organisms for regeneration, such as salamanders and zebrafish. In future work, a deeper focus on the differences in functional subpopulations of macrophages between these different species (including cell surface markers, gene expression analysis, and potential variants), will help us further understand the unique contribution of macrophages in tissue regeneration.

## Additional Information and Declarations

### Data Availability

The following information was supplied regarding data availability: This is a literature review and does not have raw data.
